# Post-traumatic stress disorder among persons with HIV who engage in heavy alcohol consumption in southwestern Uganda

**DOI:** 10.1186/s12888-021-03464-z

**Published:** 2021-09-18

**Authors:** Allen Kekibiina, Julian Adong, Robin Fatch, Nneka I. Emenyonu, Kara Marson, Brian Beesiga, Sara Lodi, Winnie R. Muyindike, Moses Kamya, Gabriel Chamie, Michael G. McDonell, Judith A. Hahn

**Affiliations:** 1 Global Health Collaborative, Mbarara University of Science and Technology, Mbarara, Uganda; 2 Department of Medicine, University of California, San Francisco, CA USA; 3 Infectious Diseases Research Collaboration (IDRC), Kampala, Uganda; 4 Boston University School of Public Health, Boston, MA USA; 5 Mbarara Regional Referral Hospital, Mbarara, Uganda; 6 Elson S Floyd College of Medicine, Washington State University, Spokane, WA USA; 7 Department of Epidemiology and Biostatistics, University of California, San Francisco, CA USA

**Keywords:** Post-traumatic stress disorder (PTSD), Alcohol, HIV, Uganda

## Abstract

**Background:**

We aimed to describe the prevalence of PTSD symptoms and its associated factors in persons living with HIV (PLWH) in Uganda who engage in heavy alcohol use.

**Methods:**

We analyzed baseline data from the Drinkers Intervention to Prevent Tuberculosis study which enrolls PLWH with latent tuberculosis who engage in heavy alcohol consumption. Using the primary care Post Traumatic Stress Disorder (PTSD) screening scale from the DSM-5 (PC-PTSD-5), probable PTSD was defined as reporting ≥3 of 5 assessed symptoms. We conducted the Alcohol Use Disorders Identification Test-Consumption and assessed demographics, smoking, symptoms of depression, and spirituality/religiosity.

**Results:**

Of 421 participants enrolled from 2018 through 2020, the majority (68.2%) were male, median age was 40 years (interquartile range [IQR]: 32–47), and median AUDIT-C score was 6 [IQR: 4–8]. Half (50.1%) of the participants reported ever experiencing a traumatic event, and 20.7% reported ≥3 symptoms of PTSD. The most commonly reported PTSD symptoms in the past 1 month in the entire sample were avoidance (28.3%), nightmares (27.3%), and being constantly on guard (21.6%). In multivariable logistic regression analyses, level of alcohol use was not associated with probable PTSD (adjusted odds ratio [AOR] for each AUDIT-C point: (1.02; 95% CI: 0.92–1.14; *p* = 0.69); however, lifetime smoking (AOR 1.89; 95% CI: 1.10–3.24) and reporting symptoms of depression (AOR 1.89; 95% CI: 1.04–3.44) were independently associated with probable PTSD.

**Conclusions and recommendations:**

A history of traumatic events and probable PTSD were frequently reported among persons who engage in heavy drinking, living with HIV in Uganda. Level of alcohol use was not associated with probable PTSD in this sample of PLWH with heavy alcohol use, however other behavioral and mental health factors were associated with probable PTSD. These data highlight the high prevalence of PTSD in this group, and the need for screening and interventions for PTSD and mental health problems.

**Supplementary Information:**

The online version contains supplementary material available at 10.1186/s12888-021-03464-z.

## Introduction

Post-traumatic stress disorder (PTSD) is a significant worldwide health concern, and is highly prevalent in sub-Saharan Africa [1]. A recent meta-analysis showed that the pooled prevalence of probable current PTSD across 10 countries in sub-Saharan Africa was 22% [1]. These numbers are drastically higher than the estimated 4% prevalence of lifetime PTSD diagnosis reported in a 25-country study that included South Africa as the sole African country [2].

Sub-Saharan Africa is also disproportionately affected by the HIV/AIDS pandemic. Of the 38 million people estimated to be living with HIV/AIDS worldwide, over 70% live in sub-Saharan Africa [3]. While HIV has become a chronic illness, due to the wide spread roll out of antiretroviral therapy (ART), and mortality from AIDS greatly reduced [4], mental health issues remain common among persons living with HIV (PLWH) [5]. These issues include PTSD, depression, substance use disorders, eating disorders, and anxiety disorders, and they commonly co-occur [6, 7]. Probable PTSD occurs due to traumatic events experienced by the general population like serious accidents, natural disasters, political upheavals, and armed conflict, all which are common in sub-Saharan Africa [1]. PLWH in sub-Saharan Africa also may have increased risk of probable PTSD due to HIV-related traumatic events, such as intimate partner violence (IPV) and physical abuse [8].

Probable PTSD is associated with impaired quality of life, poor adherence with medical interventions, and high risk sexual behaviors [9, 10]. Among PLWH, probable PTSD may negatively affect HIV treatment outcomes [11, 12], adherence [13, 14], and retention in HIV care [15]. In addition, probable PTSD is further complicated by other common comorbid mental health disorders such as depression, as well as substance and alcohol use disorders [16]; among PLWH, probable PTSD commonly co-occurs with depression and alcohol use [17, 18]. Alcohol use is highly prevalent among PLWH [19] and may act as a coping mechanism for HIV-related stress [20, 21] and possibly probable PTSD.

While levels of probable PTSD in sub-Saharan Africa have been found to be high in several studies, there has been a high degree of heterogeneity in the results [1]. In addition, few studies have examined PTSD among PLWH in sub-Saharan Africa, and we are unaware of studies measuring probable PTSD among persons engaging in heavy alcohol use in this setting. Thus, we aimed to estimate the prevalence of probable PTSD in PLWH engaging in heavy alcohol use in Southwestern Uganda. Since untreated PTSD is associated with a host of negative consequences, and heavy drinking may be a response to PTSD which is not likely to be treated in low-income settings, we also sought to determine whether the level of alcohol consumption is associated with experiencing probable PTSD, and investigated other psycho-social factors as well. We conducted these investigations within a study of PLWH who engage in heavy alcohol consumption and who are co-infected with latent tuberculosis (TB) in Southwestern Uganda.

## Materials and methods

### Setting and population

The present analysis uses baseline data (up to time of this interim analysis) from an ongoing research study of PLWH who were recruited from 4 large HIV clinics (ranging between 5000 and 15,000 patients) in Southwestern Uganda. The parent study, the Drinkers Intervention to Prevent Tuberculosis (DIPT, NCT03492216) study, is a randomized controlled trial (RCT) of economic incentives. The incentives are designed to promote reduced alcohol consumption by incentivizing negative point-of-care urine tests for the short-term alcohol biomarker, ethyl glucuronide (EtG) [22], and to promote isoniazid (INH) adherence by incentivizing INH-positive point-of-care urine tests, (using the IsoScreen test) [23], among persons who engage in heavy alcohol consumption and are co-infected with HIV and TB, while receiving 6 months of INH preventive therapy. Details of the trial are described elsewhere (NCT03492216).

### Study procedures

Screening for the DIPT Study is a multi-step process. Eligibility criteria at the initial screening step includes adults (≥18 years old) living with HIV, who are fluent in Runyankole or English, have been prescribed antiretroviral therapy (ART) for at least 6 months, live within 2 h driving distance or 60 km of the study site, have no plans to move out of the clinic’s catchment area, have no history of active TB or taking TB preventive medications, and are a current self-reported drinker (prior 3 months). Exclusion criteria includes currently taking or having taken nevirapine (NVP) in the prior 2 weeks, those who are prescribed anti-convulsant medications or who have a history of recurring seizures. Eligibility further includes being positive for heavy alcohol use via the AUDIT-C (≥3 for women; ≥4 for men); positive for recent alcohol use based on a urine ethyl glucuronide (EtG) dipstick test (300 ng/mL cutoff, by Confirm Biosciences, San Diego, California); having alanine transaminase (ALT) and aspartate transaminase (AST) levels <2x the upper limit of normal; being cleared of active TB (those reporting TB symptoms); not being pregnant, and having a positive tuberculin skin test (TST) with an induration ≥5 mm 48–72 h after injection with purified protein derivative (PPD). TST was performed by study research assistants.

### Measures

Research assistants collected data electronically with an interviewer-administered survey. The interviews were held in private rooms for confidentiality and were administered in Runyankole or English according to the participants’ language preference. The study questionnaire, including the PTSD scale described below, was translated into Runyankole, back-translated to English, and the original and back-translated questionnaires were compared to assess how accurately each item was translated. Items found to be less accurately translated were discussed further to arrive at translations best mirroring the original meaning. The interview comprised of questions on demographics, alcohol use, other substance use, medications, general health status, and several psycho-social measures described below.

#### Dependent variable - Post-traumatic stress disorder

We measured probable PTSD at baseline using the primary care PTSD Screening Scale from the DSM-5 (PC-PTSD-5) [24]. This scale first screens for ever experiencing a serious traumatic event, including a serious accident or fire, a physical or sexual assault or abuse, an earthquake or flood, a war, seeing someone being killed or seriously injured, or having a loved one die through homicide or suicide. Those who answer yes are then asked about experiencing the following PTSD symptoms in the past month: (1) having nightmares about the event or thoughts about the event when one did not want to, (2) trying hard not to think about the event(s), or going out of one’s way to avoid situations that reminded one of the event(s), (3) being constantly on guard, watchful or easily startled, (4) feeling numb or detached from people, activities, or one’s surroundings, and (5) feeling guilty or unable to stop blaming oneself or others for the event(s) or any problems the event(s) may have caused. The sum of the ‘yes’ responses to the 5 questions was used to calculate a score of 0–5; probable PTSD was defined as a score ≥ 3. The PC-PTSD-5 screening tool has shown strong diagnostic accuracy [24], and can be effectively used to identify people with probable PTSD [24, 25].

#### Independent variable - Alcohol use

The Alcohol Use Disorder Identification Test-Consumption (AUDIT-C), a brief screening for heavy drinking and or active alcohol use disorder [26], was used to assess the level of drinking; we used a version modified to assess drinking in the prior 3 months [27, 28].

#### Covariates

We collected participant characteristics that included age, sex, level of education, and marital status. We assessed general health status using the first question of the Medical Outcomes Study-HIV (MOS-HIV) Health Survey that asked; In general, would you say your health is excellent, very good, good, fair, or poor? The MOS-HIV has been used before as a validated measure of health in Uganda [29]. We assessed lifetime (ever) and current (past 3 months) tobacco and other substance use. We measured social desirability reporting using the Marlowe-Crowne Social Desirability Scale (MC-SDS) [30] 28-item modified survey (possible range 0–28), as a continuous variable. Social desirability was considered as a potential confounder for probable PTSD and alcohol use. We measured symptoms of depression using the Center for Epidemiologic Studies Depression (CESD) Scale, which has 20 questions, each scaled from 0 to 3, with a positive assessment if the score was ≥16 [31, 32]. We measured spirituality/intrinsic religiosity using the Duke University Religion Index (DUREL scale [33]) and also assessed religion. Religion and spirituality were of interest as they may impact one’s response when faced with trauma. We used intrinsic religiosity/subjective religiosity (subscale 3) to measure the participants’ degree of personal religious commitment and motivation in religious matters. The scores on this scale were considered as a continuous variable.

### Statistical analyses

We included baseline data only. We described the sample characteristics using proportions for categorical variables and mean, median, interquartile range (IQR) for continuous variables. We estimated Spearman correlations between pairs of potential predictor variables to avoid including any highly correlated variables in the same multivariable models. We used unadjusted odds ratios for two variables and adjusted for multiple variables to examine associations between the predictors and probable PTSD. The final multivariable model included level of alcohol use, using the AUDIT-C score as a continuous variable and any covariates that were associated with probable PTSD in bivariate analyses at *p* < 0.10. We also conducted two sensitivity analyses of the final model: 1) to examine whether sex acts as an effect modifier for the association between alcohol use and PTSD, we included and tested for an interaction between participant sex and alcohol use in the adjusted model; 2) we excluded depression to see its impact on the association of level of alcohol use with PTSD, i.e., exploring whether it was on the causal pathway from level of alcohol use to probable PTSD.

## Results

**Fig. 1:**
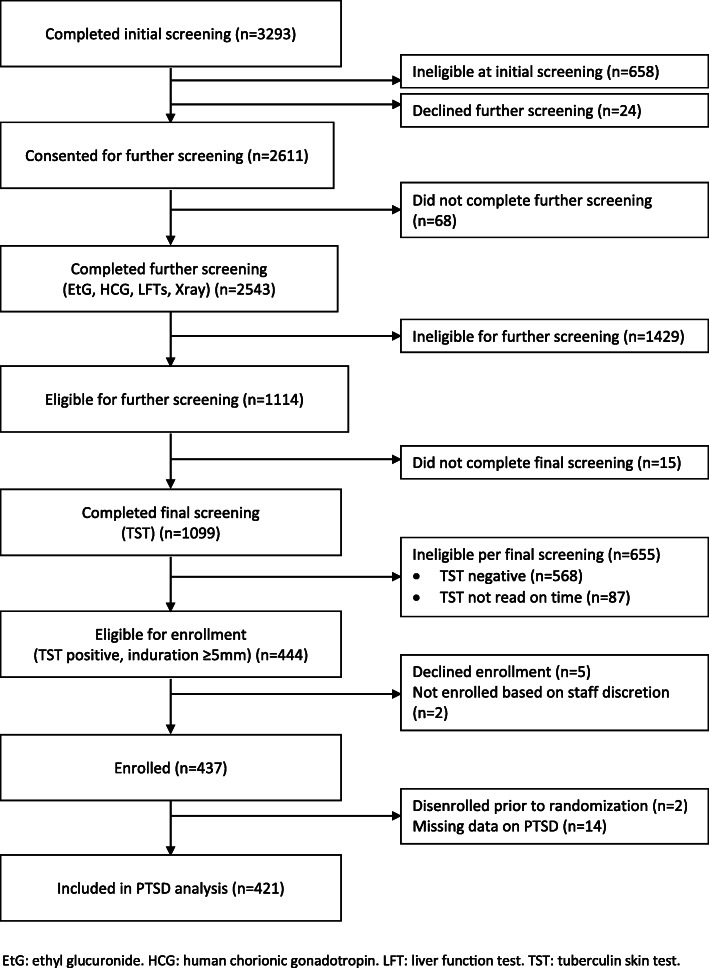
DIPT Study Screening and Enrollment through March 2020

We screened 3293 persons with HIV from May 2018 through March 2020 (Fig. 1). Of those screened, 2611 people were eligible after the initial screening step; the main reasons for exclusion at this step were having a history of taking TB medications, a history of active TB, or taking nevirapine. At the next screening step which included urine EtG screening for recent alcohol use, and testing to exclude elevated liver enzymes, active TB, and pregnancy, the most common reason for exclusion was a negative EtG test (*n* = 1251); 1114 people remained eligible for PPD screening. Finally, 435 people (39%) who tested PPD-positive, were enrolled, and participated in the baseline survey. Four hundred twenty-one (421) participants were included in this analysis, as 14 had missing answers to the PTSD questions. Of the included participants, more than two-thirds (68.2%) were male and the median age was 40 (interquartile range [IQR] 32–47). The majority of the participants reported primary school education or less (78.6%), and 59.4% of the participants were either married or cohabiting. Participants reported their religious affiliation as Catholic (50.4%), Protestant (43%) and other denominations (6.7%). One in five of the participants (18.3%) reported having depressive symptoms (CES-D ≥ 16). More than half of the participants reported their general health status was excellent or very good (52.0%). The median AUDIT-C score was 6 [IQR 4–8]. Nearly 40% of participants reported having ever smoked tobacco in their lifetime, and 12.8% of participants reported ever using illicit drugs (Table 1). Khat (a chewed stimulant) was the most commonly used illicit drug, reported by 10%, marijuana was reported by 3.8%, kuba (a Ugandan informal word for a form of smokeless tobacco) by 3.1%, petrol sniffing by 1%; while no participants reported having ever used other illicit drugs.

**Table 1:** Baseline characteristics, DIPT study participants through March 2020 with PTSD data, Uganda (*n* = 421)

	N (%) or median [IQR]
Age (years)	40 [32–47]
Sex	
Female	134 (31.8)
Male	287 (68.2)
More than a primary education?	
No	331 (78.6)
Yes	90 (21.4)
Married?	
No	171 (40.6)
Yes	250 (59.4)
Religion	
Protestant	181 (43.0)
Catholic	212 (50.4)
Other	28 (6.7)
DUREL: intrinsic religiosity	14 [10–15]
Social desirability scale	19 [17–22]
General health status	
Excellent/Very good	219 (52.0)
Good/Fair/Poor	202 (48.0)
AUDIT-C (continuous)	6 [4–8]
Lifetime smoking	
Never	261 (62.0)
Ever	160 (38.0)
Lifetime drug use	
Never	367 (87.2)
Ever	54 (12.8)
Depression (CESD ≥16)	
No	344 (81.7)
Yes	77 (18.3)
Ever experienced an unusually frightening, horrible or traumatic event?	
No	210 (49.9)
Yes	211 (50.1)
Probable PTSD? ^a^	
No	334 (79.3)
Yes	87 (20.7)
PTSD symptoms, past 1 month:	
Nightmares (=yes)	115 (27.3)
Avoidance (=yes)	119 (28.3)
On guard/easily startled (=yes)	91 (21.6)
Felt numb or detached (=yes)	43 (10.2)
Felt guilty or blamed oneself/others (=yes)	49 (11.6)

^a^ Probable PTSD? (score > =3) Respondents were considered to be having probable PTSD if they answered ‘yes’ to any 3 of the 5 questions of how the traumatic events affected them in the past month

### PTSD symptoms and prevalence of probable PTSD

Half (50.1%) of the participants reported ever experiencing a traumatic event. PTSD symptoms experienced in the past month included avoidance (28.3%), having nightmares (27.3%), feeling on guard or easily startled (21.6%), feeling guilty or blaming one’s self or others (11.6%), and feeling numb or detached (10.2%). The prevalence of probable PTSD (i.e., a score ≥ 3) among the 421 participants was 20.7% (Table 1).

In the unadjusted analyses, AUDIT-C score (OR for one unit increase 1.09; 95% CI: 0.99, 1.20) was associated with PTSD, as were age (OR = 0.96; 95% CI: 0.93, 0.98), the presence of depressive symptoms (OR = 2.37; 95% CI: 1.37, 4.09), and intrinsic religiosity (OR for one unit increase in score = 1.07; 95% CI: 1.00, 1.14) (*p* < 0.10) (Table 2). There were also associations between lifetime smoking (OR = 1.61; 95% CI 1.00, 2.59) and lifetime drug use (OR = 1.76; 95% CI: 0.93, 3.33) with probable PTSD.

**Table 2:** Unadjusted associations with and unadjusted odds ratios (OR) and (95% confidence intervals (CI)) for probable PTSD, DIPT study participants through March 2020, Uganda (n = 421)

	Probable PTSD (> = 3)		OR (95% CI)	***p***-value
	No (***n*** = 334) N (%) or median [IQR]	Yes (***n*** = 87) N (%) or median [IQR]		
AUDIT-C (continuous)	6 [4–8]	7 [4–9]	1.09 (0.99, 1.20)	0.066
Age (years)	40.5 [34–48]	37 [31–43]	0.96 (0.93, 0.98)	0.001
Sex				0.227
Female	111 (82.8)	23 (17.2)	1.00	
Male	223 (77.7)	64 (22.3)	1.39 (0.82, 2.35)	
More than a primary education?				0.861
No	262 (79.2)	69 (20.9)	1.00	
Yes	72 (80.0)	18 (20.0)	0.95 (0.53, 1.70)	
Married?				0.743
No	137 (80.1)	34 (19.9)	1.00	
Yes	197 (78.8)	53 (21.2)	1.08 (0.67, 1.76)	
Religion				0.369
Protestant	139 (76.8)	42 (23.2)	1.00	
Catholic	174 (82.1)	38 (17.9)	0.72 (0.44, 1.18)	
Other	21 (75.0)	7 (25.0)	1.10 (0.44, 2.77)	
DUREL: intrinsic religiosity	12 [9–15]	15 [11–15]	1.07 (1.00, 1.14)	0.054
General health status				0.048
Excellent/Very good	182 (83.1)	37 (16.9)	1.00	
Good/Fair/Poor	152 (75.3)	50 (24.8)	1.62 (1.00, 2.61)	
Lifetime smoking				0.050
Never	215 (82.4)	46 (17.6)	1.00	
Ever	119 (74.4)	41 (25.6)	1.61 (1.00, 2.59)	
Lifetime drug use				0.084
Never	296 (80.7)	71 (19.4)	1.00	
Ever	38 (70.4)	16 (29.6)	1.76 (0.93, 3.33)	
Depression (CESD >= 16)				0.002
No	283 (82.3)	61 (17.7)	1.00	
Yes	51 (66.2)	26 (33.8)	2.37 (1.37, 4.09)	
Social desirability score	20 [17–23]	18 [15–19]	0.85 (0.80, 0.91)	< 0.001

In adjusted analyses, self-reported level of alcohol use was not associated with probable PTSD (adjusted OR [AOR] for one unit increase in AUDIT-C point 1.02; 95% CI: 0.92, 1.14). Presence of depressive symptoms (AOR = 1.89; 95% CI: 1.04, 3.44) and lifetime smoking (AOR = 1.89; 95% CI 1.10, 3.24) were associated with probable PTSD (Table 3). There were increased odds of probable PTSD with higher levels of intrinsic religiosity (AOR for one unit increase in score 1.07; 95% CI: 0.99, 1.14) and good, fair, or poor versus excellent or very good health status (AOR = 1.54; 95% CI: 0.91, 2.61); however, these associations were not statistically significant. In sensitivity analyses, we found no significant interaction between gender and AUDIT-C level, and the association between AUDIT-C level and PTSD did not substantially change after excluding depressive symptoms (data not shown).

**Table 3:** Adjusted odds ratios (aOR) and 95% confidence intervals (CI) for probable PTSD, DIPT study participants through March 2020, Uganda (*n* = 417)

	aOR (95% CI)	p-value
AUDIT-C (continuous)	1.02 (0.92, 1.14)	0.693
Age (years)	0.96 (0.93, 0.99)	0.004
DUREL: intrinsic religiosity	1.07 (0.99, 1.14)	0.073
General health status		0.111
Excellent/Very good	1.00	
Good/Fair/Poor	1.54 (0.91, 2.61)	
Lifetime smoking		0.022
Never	1.00	
Ever	1.89 (1.10, 3.24)	
Lifetime drug use		0.722
Never	1.00	
Ever	0.88 (0.42, 1.81)	
Depression (CESD > = 16)		0.037
No	1.00	
Yes	1.89 (1.04, 3.44)	
Social desirability score	0.86 (0.80, 0.93)	< 0.001

## Discussion

In this analysis among PLWH who engage in heavy alcohol consumption in southwestern Uganda, we found that half of the participants surveyed reported experiencing a traumatic event and 21% met criteria for probable PTSD. We are aware of a handful of other studies that assessed probable PTSD among PLWH in low resource settings. The probable PTSD prevalence observed in this analysis was similar to the 19.6% prevalence of probable PTSD found in a study of PLWH in care in urban Uganda [34], lower than that observed in PLWH in a study conducted in Zimbabwe (55.3%) [35], and higher than that reported by a study among PLWH in post-conflict northern Uganda (8.3%) [9]. The prevalence we observed is quite similar to the 22% prevalence reported from a meta-analysis of data from 10 countries in sub-Saharan Africa that included some studies among PLWH, but did not calculate probable PTSD prevalence by HIV status [1]. The wide range of proportions of probable PTSD in these studies of PLWH and in the studies included in the above meta-analysis may be due to differences in scales used and differing populations. This study adds to the literature suggesting much higher rates of probable PTSD in low-resource settings in Sub-Saharan Africa compared to elsewhere [1, 36].

The level of alcohol use was not associated with probable PTSD in our study; this was consistent with the findings of a study among PLWH in a post-conflict area in northern Uganda [9]. However, we were only including people if they used alcohol at unhealthy levels, thus within this restricted sample, alcohol use severity and PTSD symptoms were not associated. But within the general population, alcohol use has been associated with probable PTSD [18, 37], and several theories have been put forward to explain this relationship. Alcohol use may lead to traumatic events that may cause probable PTSD, or may serve as self-medication to deal with symptoms of PTSD and therefore be a coping mechanism [20]. Our study population was comprised only of persons engaging in heavy drinking, so if alcohol is consumed to cope with probable PTSD up to only a certain level, e.g., a ceiling effect, such an effect might explain our failure to detect an association between level of drinking and probable PTSD. Another possible explanation for lack of association between level of drinking and probable PTSD in our sample could be underreporting of PTSD symptoms due to stigma and other barriers to admitting a mental health problem [38]. Underestimation of prevalence of mental health disorders is also another possible explanation for lack of significance between alcohol use and PTSD [39].

A notable finding was the association between depressive symptoms and probable PTSD. In our study, we found that having symptoms of depression was associated with probable PTSD; other studies of PLWH have reported similar associations [9, 40]. This association could be due to common overlapping symptoms between depression and probable PTSD [41]. Another possible explanation for this association could be the co-occurence of mental health disorders [35]. One study in South Africa that examined the prevalence and factors associated with probable PTSD found increased prevalence of depression among PLWH with probable PTSD compared to those without (29% versus 7%) [42]. Individuals with co-occurring probable PTSD and major depressive disorder tend to display more anxiety, depression and more PTSD symptoms and the occurrence is related to a more severe symptom presentation [43]. Co-existence of multiple mental disorders among PLWH is common and has been reported [35, 42]. Higher intrinsic religiosity was also associated with probable PTSD; other studies have reported religiosity as a coping mechanism for probable PTSD [44]. Those who had a history of lifetime smoking had higher odds of probable PTSD. Smoking has been found to be highly prevalent among patients with probable PTSD for its mood enhancement consequences and may serve as a coping mechanism for a cluster of negative affect, anxiety, and depression induced by probable PTSD [44–47].

## Limitations

This study was embedded in a research study that had specific entry criteria, including requiring a purified protein derivative positive (PPD+) result and being on ART for at least 6 months, thus the generalizability may be limited. We did not measure the different specific life events that may have led to probable PTSD, so we have an incomplete picture of what may have precipitated the probable PTSD. The cross-sectional design limited the findings to associations only, and we are not able to establish cause and effect between probable PTSD and factors that were associated with probable PTSD, such as depression, religiosity, and smoking. Although the populations consisted of persons with confirmed heavy alcohol use, the level of drinking was self-reported and this may be subject to social desirability. Social desirability in reporting level of alcohol use has been demonstrated in this population [48], however we attempted to correct for this by including a social desirability scale.

## Strengths

All participants who were included in this study were confirmed to be persons who engage in heavy drinking through ethyl glucuronide testing. Drinking status can be particularly unreliable by self-report due to social desirability bias, so having an objective biologic measure is a strength for measuring this. Having four different sites, two of which were in a rural setting, with extensive clinical data from a relatively large care- seeking sample, was a strength.

## Conclusion

While we found that probable PTSD is common among PLWH who engage in heavy alcohol consumption, we did not find a relationship between higher level of alcohol consumed and probable PTSD. The high level of probable PTSD and its association with depression suggests that there is a need for routine mental health screening in this population and more studies to assess coping mechanisms and design interventions for probable PTSD in this population.

## Supplementary Information

**Additional file 1.**

## Data Availability

The data and materials are available on request from the corresponding author (JH).

## Declarations

We confirm that all methods were carried out in accordance with relevant guidelines and regulations. We confirm that informed consent was obtained from study participants.
