# Supplementary material for: Post-traumatic stress disorder among persons with HIV who engage in heavy alcohol consumption in southwestern Uganda
Source: BMC Psychiatry. 2021 Sep 18;21:457. doi: 10.1186/s12888-021-03464-z (PMC8449437; doi:10.1186/s12888-021-03464-z)
Supplement: Supplementary file 1 — Additional file 1. [file 12888_2021_3464_MOESM1_ESM.pdf]

DIPT Questionnaire

A. Demographics
    Demographics (BL only)
        Daily wage and expenditure questions to be asked at all interviews
B. Alcohol use
    Lifetime Drinking (BL only)
    AUDIT Questionnaire- Full (prior year) (BL only)
    DSM-5 Alcohol Use Disorder (BL only)
    AUDIT-C (3 months)
    Current Alcohol Consumption
    30-day Alcohol Timeline Follow-back
C. Medications
    Adherence to ART and INH (INH at 3m and 6m only)
D. Health Status:
    General health (MOS-HIV question 1)
    Pregnancy and Breastfeeding (women only)
    TB (BL only)
    Lifetime and Current Tobacco and Other Substance Use (lifetime and current use at BL only; current use also at 6m, 12m)
    Intimate Partner Violence (lifetime at BL only; prior 6m at BL, 6m, 12m)
E. Alcohol Psych Measures
    Alcohol Expectancies (AFEXS) (BL only)
F. Social Measures
    Stages of change readiness and treatment eagerness scale (SOCRATES) (BL only)
G. Mental Health and Life events:
    Social Desirability Scale (28-items) (BL only)
    CESD (BL, 6m, 12m)
    PTSD (BL, 6m, 12m)
    Religious behavior (DUREL) (BL only)
    Scales of time preferences (BL only)
H. Qualitative questions (6m and 12m only)
I. Interviewer Confidence

Note: variable names are in red italics below. Coding for variables is in parentheses next to each option (these items should not be read during the interview).

Enter participant's study ID.<br><br>studyid	DPT _ _ _ _ _ _ _
What is today's date?<br><br>dateintv	_ _/_ _/_ _ _ _<br>day/month/year
Please enter your (research assistant) initials.<br><br>intv_initials	_ _

A. Demographics

Before we get started, I would like to explain to you how the interview works. As we discussed earlier, your participation in the interview and every aspect of the study are completely voluntary. You may skip any questions that you prefer not to answer, but we would appreciate your cooperation. You may also ask me to clarify any questions if you don't understand them or decide to stop the interview at any time. Finally, all of the information that you provide for the study will be kept completely confidential. Your responses to our questions are identified only by number, never by name.

We are now going to measure your height and weight.

1. Participant's gender?<br><br>sex	☐ Male (2)<br>☐ Female (1)
2a. Participant's weight?<br><br>weight	_ _ . _ _ kg
2b. Participant's height?<br><br>height	_ _ _ . _ cm

Now I will begin asking you questions. The questions are about a lot of different things in your life. Please answer the questions as well as you can, and ask me if you are not sure what I am asking or if you need help answering a question. We will keep your answers anonymous and you may decline to answer any question. Please let me know if you need a break from answering the questions.

3a. What is your age?<br><br>age	_ _ years<br>(-8 Don't know; -9 Declined)
3b. What is your date of birth? (Select don't know as appropriate if day, month, or year is unknown)<br><br>dobday<br>dobmonth<br>dobyear	_ _ day ☐ Don't know (-9)<br>_ _ month ☐ Don't know (-9)<br>_ _ _ _ year ☐ Don't know (-9)

4. What is your religion? (Mark one selection)<br><br>religion	☐ Protestant/Anglican (1)<br>☐ Catholic (2)<br>☐ Moslem (3)<br>☐ Seventh Day Adventist (4)<br>☐ Saved/Pentecostal (5)<br>☐ None (6)<br>☐ Other (8) (specify) _ _ _ _ _ _ _ _ _ _ _<br>religionothr<br>☐ Declined (-9)
5. What is the highest level of school you completed?<br><br>educ	☐ None (1)<br>☐ P1-P6 (2)<br>☐ P7 (3)<br>☐ S1-S3 (4)<br>☐ S4 (5)<br>☐ S5 (6)<br>☐ S6 (7)<br>☐ Tertiary/Vocational (8)<br>☐ University (9)<br>☐ Postgraduate (10)<br>☐ Other (11) (specify) ___ _ _ _ _ _ _ _ _ _ _<br>educothr<br>☐ Don’t know (-8)<br>☐ Declined (-9)
6. Now I would like you to read this sentence to me. In what language do you read? (Show the appropriate literacy card to subject)<br><br>literacy	☐ Cannot read at all (1)<br>☐ Able to read only parts of a sentence (2)<br>☐ Able to read whole sentence (3)<br>☐ No card with required language (4)<br>☐ Other (5) (specify language): _ _ _ _ _ _ _<br>_ _ _ _ _ literacyothr<br>☐ Declined (-9)
7. What is the main activity or job you do to provide for your family/household- either jobs you are paid for or for which you are given other goods or services? (Mark one selection)<br><br>job	☐ Teacher (1)<br>☐ Student (2)<br>☐ Technician/artisan (plumber, electrician, welder) (3)<br>☐ Military/police/security (4)<br>☐ Selling goods – on the street (5)<br>☐ Trucker/driver/conductor (6)<br>☐ Construction worker (7)<br>☐ Housekeeper (8)<br>☐ Farmer (agro, animal husbandry, etc) (9)<br>☐ Sex worker (10)<br>☐ Local brew seller (11)<br>☐ Bar attendant (12)<br>☐ Restaurant attendant (13)<br>☐ Selling goods – from an established shop (14)<br>☐ Business person (15)

	☐ Government/clerical/secretarial (16)<br>☐ Health care worker (17)<br>☐ Mechanic (18)<br>☐ Trader (19)<br>☐ None/Unemployed (20) → (skip to question 10)<br>☐ Decline (-9)<br>☐ Other (21) (specify other: indicate if he/she receives support from family or a 'friend'): __ __ __ __ __ __ __ __ __ __ __ __ __ __ __ __ __ __ jobothr

Now I will ask you some questions about your **household**. For this study, consider your household to include **people with whom you usually live and share meals**.

8. Do you or any members of your household brew alcohol at home?<br><br>brewalc	☐ Yes (1)<br>☐ No (0)<br>☐ Don't know (-8)<br>☐ Declined (-9)
8a. If yes, who?<br><br>brewalcwho	☐ Self (1)<br>☐ Other (2)<br>☐ Don't know (-8)<br>☐ Declined (-9)
9. How much money do you <u>normally</u> make in one day of doing work and other income generating activities? Please do not count money earned by your family, or money earned as interest or from renting goods and land to others.<br><br>IF THE PARTICIPANT RESPONDS WITH A SALARY, DIVIDE BY THE NUMBER OF WORKING DAYS TO OBTAIN DAILY INCOME<br><br>dailywage	___ ___ ___ , ___ ___ ___<br>MONEY EARNED PER DAY<br>(IN SHILLINGS)<br><br>(-8 don't know; -9 declined)
10. Do members of the household own any of the following items? (Mark all that apply, including only those in working condition).<br><br>(1 = selected; 0 = not selected)	☐ Electric Iron iron<br>☐ Motorbike motorbike<br>☐ Bicycle bicycle<br>☐ Shoes other than slippers shoes<br>☐ Stove with gas or electric burners stove<br>☐ Clock clock<br>☐ Television television<br>☐ Car car<br>☐ Refrigerator or freezer fridge<br>☐ Bed bed<br>☐ Lantern lantern<br>☐ Radio radio

	☐ Mobile telephone telephone<br>☐ Sofa sofa<br>☐ Cupboard cupboard<br>☐ Mattress mattress<br>☐ None of these items possessnone<br>☐ Don't know possessdk<br>☐ Declined possessdec

Now I want to ask you some questions about your spouse (men: or spouses), if you have one.

11. What is your current marital status?<br><br>marital	☐ Married, living together (1)<br>☐ Married, not living together (2)<br>☐ Divorced/Separated (3)<br>☐ Widowed (4)<br>☐ Never married and not living together (5)<br>☐ Declined (-9)
11a. Is your husband HIV positive (women)?<br>Are any of your wives HIV positive (men)?<br><br>spousehiv	☐ Yes (1)<br>☐ No (0)<br>☐ Don't know (-8)<br>☐ Declined (-9)

(Research assistant: remember this section is about people in the household, other than the participant).

12. Now I want to ask you some questions about the other members of your household. **Not including yourself,** how many other people are currently in your household? Again, for this study consider your household to include people with whom you usually live and share meals.<br><br>nhh	__ __ members of household<br>(-8 don't know; -9 declined)

Now I am going to ask you some questions about your **house**. For this study, we define your house as **the place where your household spends the most time and where you usually share meals**.

13. How many separate rooms are in your house, including all living areas, bathrooms and any other rooms?<br><br>nrooms	__ __ rooms<br>(-8 don't know; -9 declined)
14. Does your house have electricity?<br><br>electricity	☐ Yes (1)<br>☐ No (0)<br>☐ Don't know (-8)<br>☐ Declined (-9)

15. What kind of toilet facility does your house use?<br><br>wc	☐ Covered pit latrine (1)<br>☐ Pan/bucket (2)<br>☐ VIP pit latrine (pit latrine with a vent) (3)<br>☐ Uncovered pit latrine (4)<br>☐ Flush toilet (5)<br>☐ None (6)<br>☐ Other (7) (specify) _ _ _ _ _ _ _ _ _ _ _<br>wcothr<br>☐ Don't know (-8)<br>☐ Declined (-9)
16. What are the walls of your house made of?<br><br>wall	☐ Mud/dirt (1)<br>☐ Mud/clay bricks (2)<br>☐ Cement (3)<br>☐ Other (4) (specify) _ _ _ _ _ _ _ _ _ _ _<br>wallothr<br>☐ Don't know (-8)<br>☐ Declined (-9)
17. What is the floor of your housing made of?<br><br>floor	☐ Mud/dirt/dung (1)<br>☐ Mud/dirt with covering (2)<br>☐ Cement (3)<br>☐ Vinyl/asphalt strips (4)<br>☐ Tiles (5)<br>☐ Other (6) (specify) _ _ _ _ _ _ _ _ _ _ _<br>floorothr<br>☐ Don't know (-8)<br>☐ Declined (-9)
18. Where do you get water?<br><br>water	☐ Piped into dwelling/compound (1)<br>☐ Communal tap (2)<br>☐ Open well (3)<br>☐ Protected well (4)<br>☐ Protected stream – a constant flow of water out of an open pipe, in which the source is underground or protected (5)<br>☐ Unprotected spring – a constant flow of water from an open natural spring without fencing to protect it from animals or children (6)<br>☐ Stream- flowing water without a point source (7)<br>☐ Public borehole (8)<br>☐ Other (9) (specify) _ _ _ _ _ _ _ _ _ _ _<br>waterothr<br>☐ Don't know (-8)<br>☐ Declined (-9)

19. What mode of travel would you normally use to go to the nearest health facility?<br><br>modetravelhf	☐ Walk (1)<br>☐ Your own bicycle (2)<br>☐ Your own motorcycle (3)<br>☐ Your own car/truck (4)<br>☐ Animal drawn cart (5)<br>☐ Boda boda (6)<br>☐ Bus/Taxi (7)<br>☐ Special hire (8)<br>☐ Other (9)<br>Specify: ____________________<br>modetravelhf_oth<br><br>☐ Don't know (-8)
20. How long would it take you to travel to the nearest health facility with the means specified above?<br><br>timetravelhf	HOURS MINUTES<br>☐☐ ☐☐<br><br>Don't know (-8)<br><br>If less than 1 hour, enter 0 in Hours<br>Enter 0-60 in Minutes

Now I'd like to ask you about the amount that you've spent on various items in the last month (30 days).

(Research assistant: complete column 1 first and then ask column 2 for items that respondent's household purchased.)

Item name	1. In the <u>past 4 weeks</u>, have you spent any money on [ITEM]?	2. How much in total did you spend on [ITEM] in the <u>past 4 weeks</u>?<br><br>(value in Shillings)<br>(-8 Don't know; -9 Declined)
Clothing<br>clothing_yn, clothing_ush	☐ Yes →<br>☐ No (skip to next item)	____________
Footwear<br>footwear_yn, footwear_ush	☐ Yes →<br>☐ No (skip to next item)	____________
Jewelry, watches, bracelets<br>jewelry_yn, jewelry_ush	☐ Yes →<br>☐ No (skip to next item)	____________
Alcoholic beverages<br>alcoholbev_yn, alcoholbev_ush	☐ Yes →<br>☐ No (skip to next item)	____________
Non-alcoholic beverages<br>nonalcoholbev_yn, nonalcoholbev_ush	☐ Yes →<br>☐ No (skip to next item)	____________
Tobacco, cigarettes<br>tobaccocigs_yn, tobaccocigs_ush	☐ Yes →<br>☐ No (skip to next item)	____________

Toilet soap, washing powder, toothpaste, etc.<br>soap_yn, soap_ush	☐ Yes →<br>☐ No (skip to next item)	___________
Charcoal<br>charcoal_yn, charcoal_ush	☐ Yes →<br>☐ No (skip to next item)	___________
Kerosene/petrol/diesel<br>petrol_yn, petrol_ush	☐ Yes →<br>☐ No (skip to next item)	___________
Firewood<br>firewood_yn, firewood_ush	☐ Yes →<br>☐ No (skip to next item)	___________
Cooking oil<br>cookoil_yn, cookoil_ush	☐ Yes →<br>☐ No (skip to next item)	___________
Airtime for mobile phones<br>airtime_yn, airtime_ush	☐ Yes →<br>☐ No (skip to next item)	___________
Transportation<br>transport_yn, transport_ush	☐ Yes →<br>☐ No (skip to next item)	___________
Rent<br>rent_yn, rent_ush	☐ Yes →<br>☐ No (skip to next item)	___________
Electricity<br>electricity_yn, electricity_ush	☐ Yes →<br>☐ No (skip to next item)	___________
Water<br>water_yn, water_ush	☐ Yes →<br>☐ No (skip to next item)	___________
School fees/tuition/uniforms/supplies<br>schoolfees_yn, schoolfees_ush	☐ Yes →<br>☐ No (skip to next item)	___________

[men] In the <u>past 4 weeks</u>, have you given money, gifts, or favors in exchange for sex?<br>paidsex_pay<br><br>[women] In the <u>past 4 weeks</u>, have you received money, gifts, or favors in exchange for sex?<br>paidsex_receive	☐ Yes →<br>☐ No (skip to next section)	[men] How much in total value [in USh] did you give for sex in the <u>past 4 weeks</u>?<br><br>[women] How much in total value [in USh] did you receive for sex in the <u>past 4 weeks</u>?<br><br>___________

B. Alcohol Consumption

Now I have some questions about alcohol. It is important for us to know how much you take. Please try to be as honest and accurate as possible. As we told you when you agreed to participate in this study, all your answers to the questions will be kept completely confidential.

Alcohol includes **wine, beer, hard liquor, tonto, mukumbot, omuramba, kwete, malwa**, or **any beverage that contains alcohol.** The only exception to this is small amounts of communion wine or wine that you received at church or a religious ceremony. **Redbull, Rock boom, Sting, and Horsepower are not alcohol**.

Please show participant the conversion scale and other materials to help them understand drink sizes.
Conversion Scale

Wine	milliliters	ounces	drinks
Glass	140	~4.5	1
Half bottle	375	~12	~3
Bottle	750	~24	~6

Beer			
12 oz can/bottle	300-360	~12	1
500 ml can/bottle	500	~17	~1.5

Hard Liquor			
Shot	40	~1.3	1
Half pint	240	~8	~6
Pint	480	~16	~12
Fifth	750	~25	~19
Quart	1000	~32	~2

1 Standard Drink is Equal to

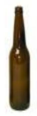

One 360 ml can/bottle of beer

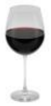

One 140 ml glass of 12% alcohol wine

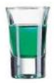

40 ml of hard liquor (e.g. waragi, rum, vodka, whiskey)

Lifetime Drinking History

1. Have you ever in your life taken alcohol?<br><br>alcever	☐ Yes (1)<br>☐ No (0) → (abstainer – skip to next section)<br>☐ Don't Know (-8) → (abstainer – skip to next section)<br>☐ Decline (-9) → (abstainer – skip to next section)
2. When was the last time you took any alcohol?<br><br>lastalc2	☐ More than 5 years ago (1)<br>☐ 1 to 5 years ago (2)<br>☐ 6 months ago – 1 year ago (7)<br>☐ 3 – 6 months ago (8)<br>☐ 3 weeks ago - 3 months ago (4)<br>☐ 4 days ago – 3 weeks ago (5)<br>☐ In the past 3 days (6)<br>☐ Don't know (-8)<br>☐ Decline (-9)

"I'm going to ask you about when you first began taking alcohol. It might be difficult to remember how much and often you drank several years back, but please try to give your best estimate."

How old were you when you began to drink at least one drink per month (regular drinking)?<br><br>alc1_age	Age at first drink __ __<br>Never → -99<br>(-8 Don't know; -9 Decline)

AUDIT

Now I'm going to ask you about alcohol you took in the last **year**.

1. In the past year, how often did you have a drink containing alcohol?<br><br>audit1_yr	☐ Never (0) → (skip to audit9a)<br>☐ Monthly or less (1)<br>☐ 2 to 4 times/month (2)<br>☐ 2 to 3 times/week (3)<br>☐ 4 or more times/week (4)<br>☐ Don't know (-8)<br>☐ Decline (-9)
2. In the past year, how many drinks containing alcohol do you have on a typical day when you are drinking?<br><br>Remind participant about drink sizes if needed: One drink is equal to, one 140 milliliter glass of wine, one 300 ml bottle of beer or two-thirds of a 500 milliliter bottle of beer, or two-thirds of a 500 ml cup of home brew, a drink with 40 milliliters of hard liquor. Show participant standard drink card.<br><br>audit2_yr	☐ None (0)<br>☐ 1 or 2 (1)<br>☐ 3 or 4 (2)<br>☐ 5 or 6 (3)<br>☐ 7 to 9 (4)<br>☐ 10 or more (5)<br>☐ Don't know (-8)<br>☐ Decline (-9)
3. In the past year, how often do you have six or more drinks on one occasion?<br><br>audit3_yr	☐ Never (0)<br>☐ Less than monthly (1)<br>☐ Monthly (2)<br>☐ Weekly (3)<br>☐ Daily or almost daily (4)<br>☐ Don't know (-8)<br>☐ Decline (-9)

4. How often during the past year have you found that you were not able to stop drinking once you had started?<br><br>audit4_yr	☐ Never (0)<br>☐ Less than monthly (1)<br>☐ Monthly (2)<br>☐ Weekly (3)<br>☐ Daily or almost daily (4)<br>☐ Don't know (-8)<br>☐ Decline (-9)
5. How often during the past year have you failed to do what was normally expected from you because of drinking?<br><br>audit5_yr	☐ Never (0)<br>☐ Less than monthly (1)<br>☐ Monthly (2)<br>☐ Weekly (3)<br>☐ Daily or almost daily (4)<br>☐ Don't know (-8)<br>☐ Decline (-9)
6. How often during the past year have you needed a first drink in the morning to get yourself going after a heavy drinking session?<br><br>audit6_yr	☐ Never (0)<br>☐ Less than monthly (1)<br>☐ Monthly (2)<br>☐ Weekly (3)<br>☐ Daily or almost daily (4)<br>☐ Don't know (-8)<br>☐ Decline (-9)
7. How often during the past year have you had a feeling of guilt or remorse after drinking?<br><br>audit7_yr	☐ Never (0)<br>☐ Less than monthly (1)<br>☐ Monthly (2)<br>☐ Weekly (3)<br>☐ Daily or almost daily (4)<br>☐ Don't know (-8)<br>☐ Decline (-9)
8. How often during the past year have you been unable to remember what happened the night before because you had been drinking?<br><br>audit8_yr	☐ Never (0)<br>☐ Less than monthly (1)<br>☐ Monthly (2)<br>☐ Weekly (3)<br>☐ Daily or almost daily (4)<br>☐ Don't know (-8)<br>☐ Decline (-9)
9a. Have you or someone else ever been injured as a result of your drinking?<br><br>audit9a	☐ No (0) → (skip to Q10)<br>☐ Yes (1)<br>☐ Don't know (-8) → (skip to Q10)<br>☐ Decline (-9) → (skip to Q10)
9b. When did this occur?	☐ In the past 3 months (0)

audit9b	☐ In the past 3 – 12 months (1)<br>☐ Over a year ago (2)<br>☐ Don't know (-8)<br>☐ Decline (-9)
10a. Has a relative or friend or a doctor or other health worker ever in your life been concerned about your drinking or suggested you cut down?<br><br>audit10a	☐ No (0) → (skip to DSM-5)<br>☐ Yes (1)<br>☐ Don't know (-8) → (skip to DSM-5)<br>☐ Decline (-9) → (skip to DSM-5)
10b. When was this?<br><br>audit10b	☐ In the past 3 months (0)<br>☐ In the past 3 – 12 months (1)<br>☐ Over a year ago (2)<br>☐ Don't know (-8)<br>☐ Declined (-9)
10c. Who suggested you cut down? (choose all that apply)<br><br>(1 = selected; 0 = not selected)	☐ Spouse/partner(s) audit10spouse<br>☐ Your child(ren) audit10child<br>☐ Female family member(s) audit10ffam<br>☐ Male family member(s) audit10mfam<br>☐ Female friend(s) audit10ffriend<br>☐ Male friend(s) audit10mfriend<br>☐ Someone at work or school audit10wrk<br>☐ A doctor or a health care worker audit10md<br>☐ Other (specify) _ _ _ _ _ _ _ _ _ _ _ _<br>audit10othr, audit10othrtxt<br>☐ Don't Know audit10dk<br>☐ Declined audit10dec

DSM-5: Alcohol Use Disorder

(Skip over DSM-5 if someone reports no alcohol use in the prior year (audit1_yr = never))

In the past year, have you: ?

	Yes (1)	No (0)	Refused (-9)
1. Had times when you ended up drinking more, or longer, than you intended?<br><br>dsm5_1	☐	☐	☐
2. More than once wanted to cut down or stop drinking, or tried to, but couldn't?<br><br>dsm5_2	☐	☐	☐

3. Spent a lot of time drinking? Or being sick or getting over other after-effects?<br><br>dsm5_3	☐	☐	☐
4. Wanted a drink so badly you couldn't think of anything else?<br><br>dsm5_4	☐	☐	☐
5. Found that drinking—or being sick from drinking—often interfered with taking care of your home or family? Or caused job troubles? Or school problems?<br><br>dsm5_5	☐	☐	☐
6. Continued to drink even though it was causing trouble with your family or friends?<br><br>dsm5_6	☐	☐	☐
7. Given up or cut back on activities that were important or interesting to you, or gave you pleasure, in order to drink?<br><br>dsm5_7	☐	☐	☐
8. More than once gotten into situations while or after drinking that increased your chances of getting hurt (such as driving a car or a boda boda, using machinery, walking in a dangerous area, getting into a fight, or having unsafe sex)?<br><br>dsm5_8	☐	☐	☐
9. Continued to drink even though it was making you feel depressed or anxious or adding to another health problem? Or after having had a memory blackout?<br><br>dsm5_9	☐	☐	☐
10. Had to drink much more than you once did to get the effect you want? Or found that your usual number of drinks had much less effect than before?<br><br>dsm5_10	☐	☐	☐
11. Found that when the effects of alcohol were wearing off, you had withdrawal symptoms, such as trouble sleeping, shakiness, restlessness, nausea, sweating, a racing heart, or a seizure? Or sensed things that were not there?	☐	☐	☐

dsm5_11			

AUDIT-C

Now I'm going to ask you about alcohol you took in the last **3 months**.

1. In the **past 3 months**, how often did you have a drink containing alcohol?<br><br>audit1a	☐ Never (0) → (skip to next section (Medications))<br>☐ Monthly or less (1)<br>☐ 2 to 4 times/month (2)<br>☐ 2 to 3 times/week (3)<br>☐ 4 or more times/week (4)<br>☐ Don't know (-8)<br>☐ Decline (-9)
2. How many drinks containing alcohol did you have on a typical day when you were drinking in the **past 3 months?**<br><br>Remind participant about drink sizes if needed: One drink is equal to, one 140 milliliter glass of wine, one 300 ml bottle of beer or two-thirds of a 500 milliliter bottle of beer, or two-thirds of a 500 ml cup of home brew, a drink with 40 milliliters of hard liquor. Show participant standard drink card.<br><br>audit2	☐ None (0)<br>☐ 1 or 2 (1)<br>☐ 3 or 4 (2)<br>☐ 5 or 6 (3)<br>☐ 7 to 9 (4)<br>☐ 10 or more (5)<br>☐ Don't know (-8)<br>☐ Declined (-9)
3. How often did you have six or more drinks on one occasion in the **past 3 months?**<br><br>audit3	☐ Never (0)<br>☐ Less than monthly (1)<br>☐ Monthly (2)<br>☐ Weekly (3)<br>☐ Daily or almost daily (4)<br>☐ Don't know (-8)<br>☐ Declined (-9)

1. Where did you usually drink alcohol in the last 3 months?<br>(check all that apply)<br><br>(1 = selected; 0 = not selected)	☐ At parties and celebrations alcloc_party<br>☐ At home alcloc_home<br>☐ At bars/drinking establishments alcloc_bar<br>☐ At restaurants alcloc_rest<br>☐ Other (specify) __ __ __ __ __ __ __ __ __ __ __ __<br>alcloc_othr, alcloc_othrtxt<br>☐ Don't know alcloc_dk<br>☐ Decline alcloc_dec

2. What days of the week did you usually drink alcohol? (check all that apply)<br><br>(1 = selected; 0 = not selected)	☐ Monday – Thursday alcweek_mt<br>☐ Friday alcweek_fri<br>☐ Saturday alcweek_sat<br>☐ Sunday alcweek_sun<br>☐ Don't know alcweek_dk<br>☐ Declined alcweek_dec
3. With whom do you usually drink alcohol? (check all that apply)<br><br>(1 = selected; 0 = not selected)	☐ Friends alcppl_friend<br>☐ Spouse alcppl_spouse<br>☐ Other sexual partners alcppl_sex<br>☐ Neighbors/the people who live near you alcppl_neighbors<br>☐ Family alcppl_family<br>☐ Strangers alcppl_strangers<br>☐ Drink alone alcppl_alone<br>☐ Don't Know alcppl_dk<br>☐ Declined to answer alcppl_dec
4. When you drink alcohol, how many people are usually drinking with you? If you usually drink in a bar or restaurant, I mean the people who are sitting with you that you, rather than the whole place.<br><br>alcbuddies	☐ You are often the only one drinking alcohol (1)<br>☐ 1 other person (2)<br>☐ 2-4 people (3)<br>☐ 5-10 people (4)<br>☐ More than ten people (5)<br>☐ Don't Know (-8)<br>☐ Decline (-9)

Current Alcohol Consumption

Now I'm going to ask you about the different types of alcohol you may have taken in the last **3 months**.

1. In the last **3 months**, did you take the following types of alcohol? Choose all that apply<br><br>(1 = selected; 0 = not selected)	☐ Commercially bottled, homemade, or locally made wine (but not small amounts of wine taken at church) wine_any<br>☐ Commercially brewed beer cbeer_any<br>☐ Home brews or local brews, like tonto, mukombot, omuramba, kwete or others, excluding malwa or other beverages taken communally hbeer_any<br>☐ Commercially made hard liquor or spirits cspirit_any<br>☐ Locally made hard liquor or spirits such as embandure and enguli? lspirit_any<br>☐ Malwa or other beverages taken communally malwa_any

	☐ None of these types alctype_none
2. In the last **3 months**, which type of alcohol did you take most often? Choose one.<br><br>alctype_common	☐ Commercially bottled, homemade, or locally made wine (but not small amounts of wine taken at church) (1)<br>☐ Commercially brewed beer (2)<br>☐ Home brews or local brews, like tonto, mukombot, omuramba, kwete or others, excluding malwa or other beverages taken communally (3)<br>☐ Commercially made hard liquor or spirits (4)<br>☐ Locally made hard liquor or spirits such as embandure and enguli? (5)<br>☐ Malwa or other beverages taken communally (6)<br>☐ All types taken equally (7)<br>☐ Don't know (-8)
3. In the last **3 months**, how often did you become drunk or intoxicated by alcohol?<br><br>drunkfreq3m	☐ Every day or nearly every day (1)<br>☐ Three or four times a week (2)<br>☐ Once or twice a week (3)<br>☐ Two to three times a month (4)<br>☐ About once a month (5)<br>☐ Once or twice in the last 3 months (6)<br>☐ Never in the last 3 months (7)<br>☐ Don't know (-8)<br>☐ Decline (-9)

30-day Alcohol Use Timeline Follow-back

Now I'm going to ask you about your even more recent alcohol use, how much alcohol you took in the past 30 days.

In the past 30 days how many days in total did you drink any wine, beer, hard liquor, tonto, mukumbot, omuramba, kwete, malwa, or any beverage that contains alcohol? Please do not include small amounts of communion wine or wine that you received at church or a religious ceremony.<br><br>alc30d	__ __ days<br>(-8 Don't Know, -9 Decline)
How many drinks did you usually take on those days that you took any alcohol?<br><br>alc30d_typ	__ __ drinks<br>(-8 Don't Know, -9 Decline)
In the past 14 days, can you try to remember which days did you take any alcohol? Approximately how many drinks did you drink on those days?<br><br>**Note to Interviewer: Identify the days the respondent took any alcohol on the calendar, prompting about weekends, public holidays, personal celebrations, etc. to try to help the participant remember. Then ask if they know approximately how many drinks each time – refer back to the drink size definitions if needed. Fill in the number of drinks. OK to put 1.5 if the participant says 1-2.**	**Start Date** (Day 1):<br>__ __ /__ __/__ __ __ __<br>dd/mm/yyyy<br><br>**End Date** (yesterday, Day 14):<br>__ __ /__ __/__ __ __ __<br>dd/mm/yyyy<br><br>(dates filled and calendar generated automatically in CASIC)

alcfb1	alcfb2	alcfb3	alcfb4	alcfb5	alcfb6	alcfb7
**1**	**2**	**3**	**4**	**5**	**6**	**7**
alcfb8	alcfb9	alcfb10	alcfb11	alcfb12	alcfb13	alcfb14
**8**	**9**	**10**	**11**	**12**	**13**	**14**

C. Medications:

Now I'm going to ask you about your experiences with HIV testing and HIV care, including medications for HIV.

1. When did you first have a test that showed that you were infected with HIV, the AIDS virus?<br><br>Select "don't know" as appropriate for day, month, or year if it is unknown.<br><br>dhivday<br>dhivmonth<br>dhivyear	Date: _ _ / _ _ / _ _ _ _<br>day/month/year<br><br>(-9 don't know)

Anti-HIV medications

Now I am going to ask you about anti-HIV medications.

1. When did you first receive anti-HIV medication?<br><br>[If day unknown, probe for beginning, middle, or end of month]<br><br>artd_day<br>artd_month<br>artd_year	_ _ / _ _ / _ _ _ _ day/month/year<br>(-9 don't know)<br><br>☐ N/A has not yet received medications → (skip to next section) art_dateRf
2. How many pills a day of anti-HIV medications are you supposed to take (not counting Septrin and multivitamin pills).<br><br>artnpills	_ _ pills<br>(-8 Don't Know; -9 Decline)
3. Which medications are you currently taking for HIV?<br><br>art_which	☐ TDF/3TC/EFV (Tenofovir, Lamivudine, Efavirenz) (1)<br>☐ AZT/3TC/EFV (Zidovudine, Lamivudine, Efavirenz) (2)<br>☐ AZT/3TC/NVP (Zidovudine, Lamivudine, Nevirapine) (Combipack, Duovir-N) (3)<br>☐ TDF/3TC/NVP (Tenofovir, Lamivudine, Nevirapine) (4)<br>☐ ABC/3TC/NVP (Abacavir, Lamivudine, Nevirapine) (5)

	☐ AZT/3TC/LPV/r (Zidovudine, Lamivudine, Lopinavir/ritonavir) (6)<br>☐ AZT/3TC/ATV/r (Zidovudine, Lamivudine, Atazanavir/ritonavir) (7)<br>☐ TDF/3TC/LPV/r (Tenofovir, Lamivudine, Lopinavir/ritonavir) (8)<br>☐ TDF/3TC/ATV/r (Tenofovir, Lamivudine, Atazanavir/ritonavir) (9)<br>☐ ABC/3TC/LPV/r (Abacavir, Lamivudine, Lopinavir/ritonavir) (10)<br>☐ ABC/3TC/ATV/r (Abacavir, Lamivudine, Atazanavir/ritonavir) (11)<br>☐ TDF/FTC/EFV (Tenofovir, Emtricitabine, Efavirenz) (Atripla) (12)<br>☐ TDF/3TC/DTG (Tenofovir, Lamivudine, Dolutegravir) (TLD) (14)<br>☐ Other (13) (specify) _ _ _ _ _ _ _ _ _ _<br>art_othr

We understand that some people on anti-HIV medication find it difficult to take it regularly and often miss doses. You may have missed doses as well.

4. How have you been at taking your anti-HIV medication in the past 30 days?<br><br>art_rate	☐ Excellent (1)<br>☐ Very good (2)<br>☐ Good (3)<br>☐ Fair (4)<br>☐ Poor (5)<br>☐ Very poor (6)<br>☐ Don’t know (-8)<br>☐ Decline (-9)
5. People have lots of reasons for taking or not taking all their pills.<br><br>In the past 30 days, did you take all your anti-HIV medication pills every day?<br><br>artday	☐ Yes (1)<br>☐ No (0)<br>☐ Don’t Know (-8)<br>☐ Decline (-9)
6. In the past 30 days, how many days, in total, have you **not** taken all of your anti-HIV medication pills?<br><br>art30d	_ _ days<br>(-8 Don’t Know; -9 Decline)
7. VA Scale Adherence	**Example**

On the line below that ranges from 0 to 100, please show me your best guess of how many of your anti-HIV medication doses you took in the last 30 days. 0 would mean you took none of your doses, and 100 would mean you took all of your doses. We would be surprised if this was 100% for most people, e.g. 0% means you have taken no medication; 50% means you have taken half your medication; 100% means you have taken every single dose of medication.<br><br>artva	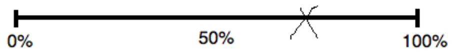<br><br>**Anti-HIV medications**<br><br>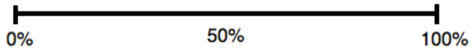
8. In the past 3 months, there been a time when you could not get your anti-HIV medications because the pharmacy was out of pills?<br><br>artnopills	☐ Yes (1)<br>☐ No (0)<br>☐ Don't know (-8)<br>☐ Decline (-9)

INH

(skipped at baseline)

Now I am going to ask you about INH, the medications you were given to prevent active TB.

1. Are you taking INH now?<br><br>inh	☐ Yes (1) → (skip to Q1c below)<br>☐ No (0)<br>☐ Don't Know (-8)<br>☐ Decline (-9)
1a. If no, why are you no longer taking INH?<br><br>inh_whystop<br><br>(1 = selected; 0 = not selected)	☐ Study doctor told me to stop inh_whystop_md<br>☐ I stopped because I didn't like how it made me feel. inh_whystop_feel<br>☐ I stopped for other reasons (specify) _<br>inh_whystop_otherreason, inh_whystopothrtxt<br>☐ I finished the course inh_whystop_finish<br>☐ Other reasons _ _ _ _ _ _ _ _ _ _ _ _ _<br>inh_whystop_othr, inh_whystopothrtxt<br>☐ Don't know inh_whystop_dk<br>☐ Declined inh_whystop_dec
1b. When is the last time you took INH?<br><br>inh_lastdday<br>inh_lastdmonth	_ _ / _ _ / _ _ _ _<br>day/month/year

inh_lastdyear	(-9 Don't Know)<br>(skip over Q1c below if not taking INH)
1c. What is motivating you to take INH?<br><br>Participants can choose/mention more than one response.<br><br>inh_motive<br><br>(1 = selected; 0 = not selected)	☐ Study doctor told me to inh_motive_doc<br>☐ Concerned about getting TB<br>inh_motive_concerntb<br>☐ I want incentives in study inh_motive_prize<br>☐ Encouragement from partner/family<br>inh_motive_fam<br>☐ Other reasons (specify)<br>__ __ __ __ __ __ __ __ __ __ __ __ __ __ __ __ __<br>inh_motive_othr inh_motive_othrtxt<br>☐ I don't know inh_motive_dk<br>☐ Declined inh_motive_dec
2. How have you been at taking your INH in the past 30 days?<br><br>inh_rate	☐ Excellent (1)<br>☐ Very good (2)<br>☐ Good (3)<br>☐ Fair (4)<br>☐ Poor (5)<br>☐ Very poor (6)<br>☐ Don't know (-8)<br>☐ Declined (-9)
3. In the past 30 days, did you take your INH pill every day?<br><br>inhday	☐ Yes (1)<br>☐ No (0)<br>☐ Don't Know (-8)<br>☐ Decline (-9)
4. In the past 30 days, how many days, in total, have you **not** taken your INH pill?<br><br>inh_30d	__ __ days<br>(-8 Don't Know; -9 Decline)
5. VA Scale Adherence - INH<br><br>On the line below that ranges from 0 to 100, please show me your best guess of how many of your INH medication doses you took in the last 30 days. 0 would mean you took none of your doses, and 100 would mean you took all of your doses. We would be surprised if this was 100% for most people, e.g. 0% means you have taken no medication; 50% means you have taken half your medication; 100% means you have taken every single dose of medication.	**Example**<br>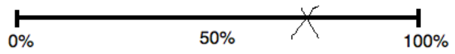<br>**INH**<br>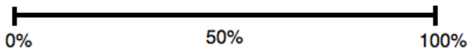

inhva	

D. Health Status:

General health

Now I have some more questions about your health.

1. In general, would you say your health is excellent, very good, good, fair, or poor?<br><br>vr12_srsi	☐ Excellent (1)<br>☐ Very good (2)<br>☐ Good (3)<br>☐ Fair (4)<br>☐ Poor (5)<br>☐ Refused (-9)

Pregnancy and breastfeeding

(skip over these questions if male)

1. Are you currently pregnant?<br><br>pregnant	☐ Yes (1)<br>☐ No (0) → (skip to TB questions)<br>☐ Don't know (-8) → (skip to TB questions)<br>☐ Declined (-9) → (skip to TB questions)
1a. How many months pregnant are you?<br><br>pregnant_mos	_____ Months<br>(-8 Don't Know, -9 Decline)
2. Are you currently breastfeeding?<br><br>breastfeed	☐ Yes (1)<br>☐ No (0)<br>☐ Don't know (-8)<br>☐ Declined (-9)

Tuberculosis diagnosis and treatment

I will now ask you some questions about tuberculosis.

1. Have you EVER known anyone who had active TB disease?<br><br>tb_others	☐ Yes (1)<br>☐ No (0)→ (skip to next section)<br>☐ Don't know (-8)→ (skip to next section)<br>☐ Declined (-9)→ (skip to next section)
2. Approximately how many people have you ever known who had active TB or TB disease?	__ __ people

tb_nothers	☐ Refused tb_nothersrf
2a. Did you share a household with any of these people? OR Do you live with any of these people?<br><br>tb_nothers_hh	☐ Yes (1)<br>☐ No (0)

Lifetime And Current Tobacco And Other Substance Use

(lifetime for baseline questionnaire only; current for baseline, 6 months, 12 months)

Now I have some questions for you about tobacco and other substances. We ask everyone these questions even if they don't use tobacco or drugs. If you've never heard of a drug that I ask you about, let me know.

	Have you **ever in your life** used any of the following:<br><br>(if no, go to next substance)	if yes:<br>Have you **ever in your life injected it** into your veins?<br><br>(cocaine, heroin, speed only)	if ever used:<br>Have you **used** this substance **in the past 3 months?**<br><br>(if no, go to next substance)	if used in the past 90 days:<br>Have you **injected** substance **in the past 3 months?**<br><br>(cocaine, heroin, speed only)	if used in the past 90 days:<br>Days used in the past 30 days?
Smoking tobacco (cigarettes)<br><br>cigs_life<br>cigs_90d<br>cigs_n30d	☐ Yes (1)<br>☐ No (0)<br>☐ Don't know (-8)<br>☐ Decline (-9)	→	☐ Yes (1)<br>☐ No (0)<br>☐ Don't know (-8)<br>☐ Decline (-9)	→	__ __
Chewing tobacco<br><br>tob_life<br>tob_90d<br>tob_n30d	☐ Yes (1)<br>☐ No (0)<br>☐ Don't know (-8)<br>☐ Decline (-9)	→	☐ Yes (1)<br>☐ No (0)<br>☐ Don't know (-8)<br>☐ Decline (-9)	→	__ __
E-cigarette (electronic cigarettes, smokeless cigarettes, vaping)<br><br>ecigs_life<br>ecigs_90d<br>ecigs_n30d	☐ Yes (1)<br>☐ No (0)<br>☐ Don't know (-8)<br>☐ Decline (-9)	→	☐ Yes (1)<br>☐ No (0)<br>☐ Don't know (-8)<br>☐ Decline (-9)	→	__ __

	Have you **ever in your life** used any of the following:<br><br>(if no, go to next substance)	if yes:<br>Have you **ever in your life injected it** into your veins?<br><br>(cocaine, heroin, speed only)	if ever used:<br>Have you **used** this substance **in the past 3 months?**<br><br>(if no, go to next substance)	if used in the past 90 days:<br>Have you **injected** substance **in the past 3 months?**<br><br>(cocaine, heroin, speed only)	if used in the past 90 days:<br>Days used in the past 30 days?
Mairunji/Mairungi (Khat)<br><br>khat_life<br>khat_90d<br>khat_n30d	☐ Yes (1)<br>☐ No (0)<br>☐ Don't know (-8)<br>☐ Decline (-9)	→	☐ Yes (1)<br>☐ No (0)<br>☐ Don't know (-8)<br>☐ Decline (-9)	→	__ __
Marijuana<br><br>mjn_life<br>mjn_90d<br>mjn_n30d	☐ Yes (1)<br>☐ No (0)<br>☐ Don't know (-8)<br>☐ Decline (-9)	→	☐ Yes (1)<br>☐ No (0)<br>☐ Don't know (-8)<br>☐ Decline (-9)	→	__ __
Hookah<br><br>hookah_life<br>hookah_90d<br>hookah_n30	☐ Yes (1)<br>☐ No (0)<br>☐ Don't know (-8)<br>☐ Decline (-9)	→	☐ Yes (1)<br>☐ No (0)<br>☐ Don't know (-8)<br>☐ Decline (-9)	→	__ __
Powder (kuba)<br><br>powder_life<br>powder_90d<br>powder_n30d	☐ Yes (1)<br>☐ No (0)<br>☐ Don't know (-8)<br>☐ Decline (-9)	→	☐ Yes (1)<br>☐ No (0)<br>☐ Don't know (-8)<br>☐ Decline (-9)	→	__ __
Kerosene, petrol, glue, or paint thinner (for sniffing)<br><br>gas_life<br>gas_90d	☐ Yes (1)<br>☐ No (0)<br>☐ Don't know (-8)<br>☐ Decline (-9)	→	☐ Yes (1)<br>☐ No (0)<br>☐ Don't know (-8)<br>☐ Decline (-9)	→	→
Cocaine<br><br>coc_life<br>coc_inj<br>coc_90d<br>coc_inj90d	☐ Yes (1)<br>☐ No (0)<br>☐ Don't know (-8)<br>☐ Decline (-9)	☐ Yes (1)<br>☐ No (0)<br>☐ Don't know (-8)<br>☐ Decline (-9)	☐ Yes (1)<br>☐ No (0)<br>☐ Don't know (-8)<br>☐ Decline (-9)	☐ Yes (1)<br>☐ No (0)<br>☐ Don't know (-8)<br>☐ Decline (-9)	→
Heroin	☐ Yes (1)<br>☐ No (0)	☐ Yes (1)<br>☐ No (0)	☐ Yes (1)<br>☐ No (0)	☐ Yes (1)<br>☐ No (0)	→

	Have you **ever in your life** used any of the following:<br><br>(if no, go to next substance)	if yes:<br>Have you **ever in your life injected it** into your veins?<br><br>(cocaine, heroin, speed only)	if ever used:<br>Have you **used** this substance **in the past 3 months?**<br><br>(if no, go to next substance)	if used in the past 90 days:<br>Have you **injected** substance **in the past 3 months?**<br><br>(cocaine, heroin, speed only)	if used in the past 90 days:<br>Days used in the past 30 days?
hern_life<br>hern_inj<br>hern_90d<br>hern_inj90d	☐ Don't know (-8)<br>☐ Decline (-9)	☐ Don't know (-8)<br>☐ Decline (-9)	☐ Don't know (-8)<br>☐ Decline (-9)	☐ Don't know (-8)<br>☐ Decline (-9)	
Speed (Methamphetamine)<br><br>spd_life<br>spd_inj<br>spd_90d<br>spd_inj90d	☐ Yes (1)<br>☐ No (0)<br>☐ Don't know (-8)<br>☐ Decline (-9)	☐ Yes (1)<br>☐ No (0)<br>☐ Don't know (-8)<br>☐ Decline (-9)	☐ Yes (1)<br>☐ No (0)<br>☐ Don't know (-8)<br>☐ Decline (-9)	☐ Yes (1)<br>☐ No (0)<br>☐ Don't know (-8)<br>☐ Decline (-9)	→

Intimate Partner Violence (IPV)

[Note to RA: lifetime IPV questions only asked at baseline]

People in relationships often disagree and sometimes these fights become physical. I am going to ask some questions about this. I want you to speak freely and remember that everything you say will be confidential.

1. [Read all options before pausing] Have any of your sexual partners ever done any of the following:<br><br>Pushed, pulled, slapped, or held you down? Punched you? Kicked you or dragged you? Tried to strangle or burn you? Threatened or attacked you with a gun/knife/other weapon?	☐ Yes (1)<br>☐ No (0) → (skip to IPV Q2)<br>☐ Don't Know (-8) → (skip to IPV Q2)<br>☐ Decline (-9) → (skip to IPV Q2)
1a. Has this happened in the past 6 months?	☐ Yes (1)<br>☐ No (0)<br>☐ Don't Know (-8)<br>☐ Decline (-9)

2. [Read all options before pausing] Have you ever physically hurt or threatened a sexual partner, including:<br><br>Pushed, pulled, slapped, or held him/her down? Punched him/her? Kicked or dragged him/her? Tried to strangle or burn him/her? Threatened or attacked him/her with a gun/knife/other weapon?	☐ Yes (1)<br>☐ No (0) → (skip to IPV Q3)<br>☐ Don't Know (-8) → (skip to IPV Q3)<br>☐ Decline (-9) → (skip to IPV Q3)
2a. Has this happened in the past 6 months?	☐ Yes (1)<br>☐ No (0)<br>☐ Don't Know (-8)<br>☐ Decline (-9)
3. Has anyone ever physically forced you to have sex with him/her when you did not want to?	☐ Yes (1)<br>☐ No (0) → (skip to IPV Q4)<br>☐ Don't Know (-8) → (skip to IPV Q4)<br>☐ Decline (-9) → (skip to IPV Q4)
3a. Has this happened in the past 6 months?	☐ Yes (1)<br>☐ No (0)<br>☐ Don't Know (-8)<br>☐ Decline (-9)
4. Have you ever physically forced anyone to have sex with you when he/she did not want to?	☐ Yes (1)<br>☐ No (0) → (skip to next section)<br>☐ Don't Know (-8) → (skip to next section)<br>☐ Decline (-9) → (skip to next section)
4a. Has this happened in the past 6 months?	☐ Yes (1)<br>☐ No (0)<br>☐ Don't Know (-8)<br>☐ Decline (-9)

E. Alcohol Psych Measures

Alcohol Expectancy Scale – Revised

SKIP PATTERN: Skip these questions if the answer to AUDIT question 1a is "Never" (Never took a drink containing alcohol in the past year)

Many people believe that alcohol can influence how they feel and act. We would like to know how <u>you</u> think drinking alcohol affects <u>your</u> feelings and behavior.<br><br>I am going to read you a few statements that refer to how you feel when you drink alcohol. Please indicate the extent to which you agree or disagree with each statement.<br><br>When I drink alcohol…								
	Strongly disagree (1)	Disagree (2)	Slightly disagree (3)	Slightly agree (4)	Agree (5)	Strongly agree (6)	Don't know (-8)	Declined (-9)
4. I am more likely to do things I normally wouldn't do<br>ae3	☐	☐	☐	☐	☐	☐	☐	☐
6. I feel sick the next day<br>ae5	☐	☐	☐	☐	☐	☐	☐	☐
8. I feel sick (at the time I'm drinking)<br>ae6	☐	☐	☐	☐	☐	☐	☐	☐
12. I become more extroverted<br>ae7	☐	☐	☐	☐	☐	☐	☐	☐
18. I become more aggressive<br>ae10	☐	☐	☐	☐	☐	☐	☐	☐
7. I feel closer to a sexual partner<br>ae11	☐	☐	☐	☐	☐	☐	☐	☐
9. I am less nervous about sex<br>ae12	☐	☐	☐	☐	☐	☐	☐	☐
10. I find it harder to say no to sexual advances<br>ae13	☐	☐	☐	☐	☐	☐	☐	☐
11. I am a better lover<br>ae14	☐	☐	☐	☐	☐	☐	☐	☐
13. I am more sexually responsive	☐	☐	☐	☐	☐	☐	☐	☐

ae15								
15. I enjoy sex more than usual<br>ae17	☐	☐	☐	☐	☐	☐	☐	☐

F. Social Measures

SOCRATES – Stages of Change Scale

I am now going to read a series of statements to you. Each one describes a way that you might (or might not) feel about your drinking. For each statement, please tell me how much you agree or disagree with it right now. Do you strongly disagree, disagree, are you undecided or unsure, agree, or strongly agree?

	**NO! Strongly Disagree (1)**	**No Disagree (2)**	**? Undecided or Unsure (3)**	**Yes Agree (4)**	**YES! Strongly Agree (5)**	**Don't know (-8)**	**Declined (-9)**
1. I really want to make changes in my drinking.	☐	☐	☐	☐	☐	☐	☐
2. Sometimes I wonder if I am an alcoholic.	☐	☐	☐	☐	☐	☐	☐
3. If I don't change my drinking soon, my problems are going to get worse.	☐	☐	☐	☐	☐	☐	☐
4. I have already started making some changes in my drinking.	☐	☐	☐	☐	☐	☐	☐
5. I was drinking too much at one time, but I've managed to change my drinking.	☐	☐	☐	☐	☐	☐	☐
6. Sometimes I wonder if my drinking is hurting other people.	☐	☐	☐	☐	☐	☐	☐
7. I am a problem drinker.	☐	☐	☐	☐	☐	☐	☐
8. I'm not just thinking about changing my drinking, I'm already doing something about it.	☐	☐	☐	☐	☐	☐	☐
9. I have already changed my drinking, and I am looking for ways to keep from slipping back to my old pattern.	☐	☐	☐	☐	☐	☐	☐

10. I have serious problems with drinking.	☐	☐	☐	☐	☐	☐	☐
11. Sometimes I wonder if I am in control of my drinking.	☐	☐	☐	☐	☐	☐	☐
12. My drinking is causing a lot of harm.	☐	☐	☐	☐	☐	☐	☐
13. I am actively doing things now to cut down or stop drinking.	☐	☐	☐	☐	☐	☐	☐
14. I want help to keep from going back to the drinking problems that I had before.	☐	☐	☐	☐	☐	☐	☐
15. I know that I have a drinking problem.	☐	☐	☐	☐	☐	☐	☐
16. There are times when I wonder if I drink too much.	☐	☐	☐	☐	☐	☐	☐
17. I am an alcoholic.	☐	☐	☐	☐	☐	☐	☐
18. I am working hard to change my drinking.	☐	☐	☐	☐	☐	☐	☐
19. I have made some changes in my drinking, and I want some help to keep from going back to the way I used to drink.	☐	☐	☐	☐	☐	☐	☐

G. Mental Health

Social Desirability Scale

(28-items from Vu 2011)

Now I am going to read a number of statements concerning personal attitudes and traits. Please decide whether each statement is true or false as it relates to you personally.

	True (1)	False (2)	Don't Know (-8)	Decline (-9)
1. It is sometimes hard for me to go on with my work if I am not encouraged.<br>sds1	☐	☐	☐	☐
2. I sometimes feel resentful when I don't get my way.<br>sds2	☐	☐	☐	☐
3. On a few occasions, I have given up doing something because I thought I couldn't do it.<br>sds3	☐	☐	☐	☐
4. There have been times when I felt like rebelling against people in charge even though I knew they were right.<br>sds4	☐	☐	☐	☐
5. No matter whom I'm talking to, I'm always a good listener.<br>sds5	☐	☐	☐	☐
6. There have been times when I used someone for my own benefit.<br>sds6	☐	☐	☐	☐
7. I'm always willing to admit it when I make a mistake.<br>sds7	☐	☐	☐	☐
8. I sometimes try to get even, rather than forgive and forget.<br>sds8	☐	☐	☐	☐
9. I am always courteous, even to people who are disagreeable.<br>sds9	☐	☐	☐	☐
10. I have never been annoyed when people expressed ideas very different from my own.<br>sds10	☐	☐	☐	☐
11. There have been times when I was quite jealous of the good luck of others.<br>sds11	☐	☐	☐	☐

12. I am sometimes irritated by people who ask favors of me.<br>sds12	☐	☐	☐	☐
13. I have never deliberately said something that hurt someone's feelings.<br>sds13	☐	☐	☐	☐
14. I never hesitate to go out of my way to help someone who is in trouble.<br>sds14	☐	☐	☐	☐
15. I have never intensely disliked anyone.<br>sds15	☐	☐	☐	☐
16. On occasion I had doubts about my ability to succeed in life.<br>sds16	☐	☐	☐	☐
17. I am always careful about my manner of dress.<br>sds17	☐	☐	☐	☐
18. I like to gossip at times.<br>sds18	☐	☐	☐	☐
19. I can remember "playing sick" to get out of something.<br>sds19	☐	☐	☐	☐
20. I always try to practice what I preach.<br>sds20	☐	☐	☐	☐
21. I don't find it particularly difficult to get along with loud mouthed, obnoxious people.<br>sds21	☐	☐	☐	☐
22. When I don't know something, I don't at all mind admitting it.<br>sds22	☐	☐	☐	☐
23. At times I have really insisted on having things my own way.<br>sds23	☐	☐	☐	☐
24. There have been occasions when I felt like smashing things.<br>sds24	☐	☐	☐	☐
25. I would never think of letting someone else be punished for my wrongdoings.<br>sds25	☐	☐	☐	☐
26. I never resent being asked to return a favor.<br>sds26	☐	☐	☐	☐
27. I have never felt that I was punished without cause.<br>sds27	☐	☐	☐	☐
28. I sometimes think when people have a misfortune they only got what they deserved.<br>sds28	☐	☐	☐	☐

CESD

Now I am going to ask you about different ways you might have felt or behaved. Please tell me how often you have felt this way during **the past week**.

During the past week, …	Rarely or none of the time (less than 1 day) (0)	Some or a little of the time (1-2 days) (1)	Occasionally or a moderate amount of the time (3-4 days) (2)	Most or all of the time (5-7 days) (3)	Don't know (-8)	Declined (-9)
1. I was bothered by things that usually don't bother me. cesd1	☐	☐	☐	☐	☐	☐
2. I did not feel like eating; my appetite was poor. cesd2	☐	☐	☐	☐	☐	☐
3. I felt that I could not shake off the blues, even with help from my family or friends. cesd3	☐	☐	☐	☐	☐	☐
4. I felt I was just as good as other people. cesd4	☐	☐	☐	☐	☐	☐
5. I had trouble keeping my mind on what I was doing. cesd5	☐	☐	☐	☐	☐	☐
6. I felt depressed. cesd6	☐	☐	☐	☐	☐	☐
7. I felt that everything I did was an effort. cesd7	☐	☐	☐	☐	☐	☐
8. I felt hopeful about the future. cesd8	☐	☐	☐	☐	☐	☐
9. I thought my life had been a failure. cesd9	☐	☐	☐	☐	☐	☐
10. I felt fearful. cesd10	☐	☐	☐	☐	☐	☐
11. My sleep was restless. cesd11	☐	☐	☐	☐	☐	☐
12. I was happy.	☐	☐	☐	☐	☐	☐

cesd12						
13. I talked less than usual.<br>cesd13	☐	☐	☐	☐	☐	☐
14. I felt lonely.<br>cesd14	☐	☐	☐	☐	☐	☐
15. People were unfriendly.<br>cesd15	☐	☐	☐	☐	☐	☐
16. I enjoyed life.<br>cesd16	☐	☐	☐	☐	☐	☐
17. I had crying spells.<br>cesd17	☐	☐	☐	☐	☐	☐
18. I felt sad.<br>cesd18	☐	☐	☐	☐	☐	☐
19. I felt that people disliked me.<br>cesd19	☐	☐	☐	☐	☐	☐
20. I could not get “going”.<br>cesd20	☐	☐	☐	☐	☐	☐

PTSD

1. Sometimes things happen to people that are unusually or especially frightening, horrible or traumatic.<br><br>For example:<br>- A serious accident or fire<br>- A physical or sexual assault or abuse<br>- An earthquake or flood<br>- A war<br>- Seeing someone be killed or seriously injured<br>- Having a loved one die through homicide or suicide<br><br>Have you ever experienced this kind of event?<br><br>ptsd_event	☐ Yes (1)<br>☐ No (0) (→ skip to next section)<br>☐ Don't know (-8) (→ skip to next section)<br>☐ Decline (-9) (→ skip to next section)

In the past month, have you…	Yes (1)	No (0)	Don't Know (-8)	Decline (-9)
1. had nightmares about the event(s) or thought about the event(s) when you did not want to?<br><br>ptsd_nightmare	☐	☐	☐	☐
2. tried hard not to think about the event(s) or went out of your way to avoid situations that reminded you of the event(s)?<br><br>ptsd_avoid	☐	☐	☐	☐
3. been constantly on guard, watchful, or easily startled?<br><br>ptsd_guard	☐	☐	☐	☐
4. felt numb or detached from people, activities, or your surroundings?<br><br>ptsd_numb	☐	☐	☐	☐
5. felt guilty or unable to stop blaming yourself or others for the event(s) or any problems the event(s) may have caused?<br><br>ptsd_guilt	☐	☐	☐	☐

Spirituality/Religiosity: The Duke University Religion Index (DUREL)

I am going to read some statements to you about spirituality and religious activities.

1. How often do you attend church, mosque, or other religious meetings?<br><br>durel_attend	☐ Never (1)<br>☐ Once a year or less (2)<br>☐ A few times a year (3)<br>☐ A few times a month (4)<br>☐ Once a week (5)<br>☐ More than once a week (6)<br>☐ Don't know (-8)<br>☐ Declined (-9)
2. How often do you spend time in private religious activities, such as prayer, meditation or Bible/Koran study?<br><br>durel_private	☐ Rarely or never (1)<br>☐ A few times a month (2)<br>☐ Once a week (3)<br>☐ Two or more times a week (4)<br>☐ Daily (5)<br>☐ More than once a day (6)<br>☐ Don't know (-8)<br>☐ Decline (-9)

The following section contains 3 statements about religious belief or experience. Please tell me the extent to which each statement is true or not true for you – is it: Definitely not true; Tends not to be true; Unsure; Tends to be true; or Definitely true of you?

	Definitely not true (1)	Tends not to be true (2)	Unsure (3)	Tends to be true (4)	Definitely true of me (5)	Don't know (-8)	Declined (-9)
3. In my life, I experience the presence of the Divine (i.e., God)<br><br>durel_divine	☐	☐	☐	☐	☐	☐	☐
4. My religious beliefs are what really lie behind my whole approach to life<br><br>durel_beliefs	☐	☐	☐	☐	☐	☐	☐
5. I try hard to carry my religion over into all other dealings in life<br><br>durel_religion	☐	☐	☐	☐	☐	☐	☐

Time and risk preferences

Time preferences (part 1)

The next set of questions will ask you to imagine that you have won a prize, and will ask you to make decisions about when to receive the prize money. These questions are only hypothetical (pretend). You will not be receiving this prize money.

Suppose that you have just won a prize that will give you a series of payments over time. You can select the prize from two options: receiving a certain amount of money now, or waiting 14 days to receive a little more money.

Please indicate your preferred option for various scenarios below (1=Now; 2=Wait).

Research assistant: ask the respondent to imagine that the person giving you the money is someone you trust, so if the respondent chooses to wait, they will indeed receive the money in the future.

1. Would you prefer 30,000 USh now or 35,000 USh in 14 days?<br><br>time_pref1	☐ Now (1)<br>☐ Wait (2) (→ skip to next section)
2. Would you prefer 30,000 USh now or 40,000 USh in 14 days?<br><br>time_pref2	☐ Now (1)<br>☐ Wait (2) (→ skip to next section)
3. Would you prefer 30,000 USh now or 45,000 USh in 14 days<br><br>time_pref3	☐ Now (1)<br>☐ Wait (2) (→ skip to next section)
4. Would you prefer 30,000 USh now or 50,000 USh in 14 days?<br><br>time_pref4	☐ Now (1)<br>☐ Wait (2) (→ skip to next section)
5. Would you prefer 30,000 USh now or 60,000 USh in 14 days?<br><br>time_pref5	☐ Now (1)<br>☐ Wait (2) (→ skip to next section)
6. Would you prefer 30,000 USh now or 70,000 USh in 14 days?<br><br>time_pref6	☐ Now (1)<br>☐ Wait (2) (→ skip to next section)
7. Would you prefer 30,000 USh now or 80,000 USh in 14 days?<br><br>time_pref7	☐ Now (1)<br>☐ Wait (2) (→ skip to next section)

8. Would you prefer 30,000 USh now or 90,000 USh in 14 days?<br><br>time_pref8	☐ Now (1)<br>☐ Wait (2) (→ skip to next section)

Risk preferences:

The next set of questions will ask you to imagine that you have 30,000 USh of starting money, and will ask you to choose whether or not to make an investment with that money. You will know the chances of making money or losing money from this investment. Then you will be asked whether you want to invest all of the money, part of it, or none of it.

For example, say you were to invest 30,000 USh in tomatoes. If you sold them all, you would receive 90,000 USh, but if they went rotten before you could sell any, you would lose your 30,000 USh. Investing in tomatoes therefore has a certain risk—you could make money or you could lose money. Specifically, this investment will give you back 3 times the amount you invest ½ of the time and will give you back nothing ½ of the time. Do you understand what I mean when I say ½ of the time? This is the chance that if I were to flip a coin in the air, that it would come up heads.

How much of 30,000 USh would you like to invest in buying tomatoes if half the time you would make <u>3 times</u> what you bought them for, and half the time they would go rotten before you could sell any of them and you would make nothing? This means that ½ the time the investment is tripled (multiplied by 3) and ½ the time you lose your investment. I am going to read you your options. Please tell me which you choose.

riskpref

	**You keep**	**You invest**	**½ the time the investment works out and you get from the investment**	**½ the time the investment fails and you get from the investment**	**Your choice**
1	30,000	0	0	0	☐
3	25,000	5,000	3x5,000=15,000	0	☐
4	20,000	10,000	3x10,000=30,000	0	☐
5	15,000	15,000	3x15,000=45,000	0	☐
6	10,000	20,000	3x20,000=60,000	0	☐
7	5,000	25,000	3x25,000=75,000	0	☐
8	0	30,000	3x30,000=90,000	0	☐

Time preferences (part 2)

The next set of questions will ask you again to imagine that you have won a prize, and will ask you to make decisions about when to receive the prize money. Again, these questions are only hypothetical (pretend). You will not be receiving this prize money.

Suppose that you have just won a prize that will give you a series of payments over time. You can select the prize from two options: receiving a certain amount of money in 14 days, or waiting till 1 month to receive a little more money.

Please indicate your preferred option for various scenarios below (1=Wait 14 days; 2=Wait 1 month). Interviewer: ask the respondent to imagine that the person giving you the money is someone you trust, so they will indeed receive the money in the future.

1. Would you prefer 30,000 USh 14 days or 35,000 USh in 1 month?<br><br>time2_pref1	☐ Wait 14 days (1)<br>☐ Wait 1 month (2) (→ skip to next section)
2. Would you prefer 30,000 USh 14 days or 40,000 USh in 1 month?<br><br>time2_pref2	☐ Wait 14 days (1)<br>☐ Wait 1 month (2) (→ skip to next section)
3. Would you prefer 30,000 USh 14 days or 45,000 USh in 1 month?<br><br>time2_pref3	☐ Wait 14 days (1)<br>☐ Wait 1 month (2) (→ skip to next section)
4. Would you prefer 30,000 USh 14 days or 50,000 USh in 1 month?<br><br>time2_pref4	☐ Wait 14 days (1)<br>☐ Wait 1 month (2) (→ skip to next section)
5. Would you prefer 30,000 USh 14 days or 60,000 USh in 1 month?<br><br>time2_pref5	☐ Wait 14 days (1)<br>☐ Wait 1 month (2) (→ skip to next section)
6. Would you prefer 30,000 USh 14 days or 70,000 USh in 1 month?<br><br>time2_pref6	☐ Wait 14 days (1)<br>☐ Wait 1 month (2) (→ skip to next section)
7. Would you prefer 30,000 USh 14 days or 80,000 USh in 1 month?<br><br>time2_pref7	☐ Wait 14 days (1)<br>☐ Wait 1 month (2) (→ skip to next section)
8. Would you prefer 30,000 USh 14 days or 90,000 USh in 1 month?	☐ Wait 14 days (1)<br>☐ Wait 1 month (2) (→ skip to next section)

time2_pref8	

H. Qualitative Questions

I now have some questions to ask you about your participation in our study. We value your opinion and want to learn more about if and how this study may have affected you or your family and friends.

1a. In your opinion, has participating in this study affected you, your health or your economic situation (including your job)?<br><br>dipt_affectu	☐ Yes (1)<br>☐ No (0) (→ skip to 1c)<br>☐ Don't know (-8) (→ skip to 1c)
1b. If yes, how?<br><br>(Note to RA: allow for an open-ended question and answer first, before proceeding to questions 1c and 1d.)<br><br>dipt_affectuhow	_ _ _ _ _ _ _ _ _ _ _ _ _ _ _ _ _ _ _ _<br>_ _ _ _ _ _ _ _ _ _ _ _ _ _ _ _ _ _ _ _<br>_ _ _ _ _ _ _ _ _ _ _ _ _ _ _ _ _ _ _ _<br>_ _ _ _ _ _ _ _ _ _ _ _ _ _ _
1c. To what extent do you think participating in this study affected your alcohol use?<br><br>dipt_affectualc	_ _ _ _ _ _ _ _ _ _ _ _ _ _ _ _ _ _ _ _<br>_ _ _ _ _ _ _ _ _ _ _ _ _ _ _ _ _ _ _ _<br>_ _ _ _ _ _ _ _ _ _ _ _ _ _ _ _ _ _ _ _<br>_ _ _ _ _ _ _ _ _ _ _ _ _ _ _
1d. To what extent do you think participating in this study affected your ability to take your medication?<br><br>dipt_affectumed	_ _ _ _ _ _ _ _ _ _ _ _ _ _ _ _ _ _ _ _<br>_ _ _ _ _ _ _ _ _ _ _ _ _ _ _ _ _ _ _ _<br>_ _ _ _ _ _ _ _ _ _ _ _ _ _ _ _ _ _ _ _<br>_ _ _ _ _ _ _ _ _ _ _ _ _ _ _
2a. In your opinion, has participating in this study affected your partner, your family or your household members?<br><br>dipt_affectothr	☐ Yes (1)<br>☐ No (0) (→ skip to 3)<br>☐ Don't know (-8) (→ skip to 3)
2b. If yes, how?<br><br>(Note to RA: allow for an open-ended question and answer)<br><br>dipt_affectothrhow	_ _ _ _ _ _ _ _ _ _ _ _ _ _ _ _ _ _ _ _<br>_ _ _ _ _ _ _ _ _ _ _ _ _ _ _ _ _ _ _ _<br>_ _ _ _ _ _ _ _ _ _ _ _ _ _ _ _ _ _ _ _<br>_ _ _ _ _ _ _ _ _ _ _ _ _ _ _

3. In your opinion, have there been any benefits of being in this study (not already mentioned above)?<br><br>(Note to RA: allow for an open-ended question and answer)<br><br>dipt_benefits	_ _ _ _ _ _ _ _ _ _ _ _ _ _ _ _ _ _ _<br>_ _ _ _ _ _ _ _ _ _ _ _ _ _ _ _ _ _ _<br>_ _ _ _ _ _ _ _ _ _ _ _ _ _ _ _ _ _ _<br>_ _ _ _ _ _ _ _ _ _ _ _ _ _ _
4. In your opinion, have there been any challenges of being in this study (not already mentioned above)?<br><br>(Note to RA: allow for an open-ended question and answer)<br><br>dipt_challenges	_ _ _ _ _ _ _ _ _ _ _ _ _ _ _ _ _ _ _<br>_ _ _ _ _ _ _ _ _ _ _ _ _ _ _ _ _ _ _<br>_ _ _ _ _ _ _ _ _ _ _ _ _ _ _ _ _ _ _<br>_ _ _ _ _ _ _ _ _ _ _ _ _ _ _
Question for the RA only (do not ask the participant):<br><br>In your opinion as a DIPT study RA, have you observed any changes, either positive or negative, in this participant – either from direct observation and discussion with the participant or from discussions with the participant's family or friends – during his/her participation in the DIPT trial?<br><br>dipt_racomments	_ _ _ _ _ _ _ _ _ _ _ _ _ _ _ _ _ _ _<br>_ _ _ _ _ _ _ _ _ _ _ _ _ _ _ _ _ _ _<br>_ _ _ _ _ _ _ _ _ _ _ _ _ _ _ _ _ _ _<br>_ _ _ _ _ _ _ _ _ _ _ _ _ _ _

I. Interviewer Confidence

Research assistant please answer:

1. Overall, how clear and interpretable were the questions to the respondent?<br><br>ic_overall	☐ Very clear (1)<br>☐ Somewhat clear (2)<br>☐ Not at all clear (3)
2. Did the respondent have any trouble answering the time and risk preference questions?<br><br>ic_time	☐ Yes (1)<br>☐ No (0)
3. Notes on any questions that were problematic:<br><br>ic_problem	__ __ __ __ __ __ __ __ __ __ __ __ __ __ __ __ __ __ __<br>__ __ __ __ __ __ __ __ __ __ __ __ __ __ __ __ __ __ __<br>__ __ __ __ __ __ __ __ __ __ __ __ __ __ __ __ __ __ __<br>__ __ __ __ __ __ __ __ __ __ __ __ __ __ __<br><br>☐ No problematic questions (-7)<br>ic_problemrf
4. How confident are you that the respondent's answers are valid?<br><br>ic_valid	☐ Completely confident (1)<br>☐ Somewhat confident (2)<br>☐ Not at all confident (3)
5. Reasons for lack of validity:<br><br>ic_invalid	__ __ __ __ __ __ __ __ __ __ __ __ __ __ __ __ __ __ __<br>__ __ __ __ __ __ __ __ __ __ __ __ __ __ __ __ __ __ __<br>__ __ __ __ __ __ __ __ __ __ __ __ __ __ __ __ __ __ __<br>__ __ __ __ __ __ __ __ __ __ __ __ __ __ __<br><br>☐ No notes (-7)<br>ic_invalidrf
6. Research Assistant Notes:<br><br>(Put any information that you think is important to the study.)<br><br>ic_notes	__ __ __ __ __ __ __ __ __ __ __ __ __ __ __ __ __ __ __<br>__ __ __ __ __ __ __ __ __ __ __ __ __ __ __ __ __ __ __<br>__ __ __ __ __ __ __ __ __ __ __ __ __ __ __ __ __ __ __<br>__ __ __ __ __ __ __ __ __ __ __ __ __ __ __<br><br>☐ No notes (-7)<br>ic_notesrf
